# Directional intensified feature description using tertiary filtering for augmented reality tracking

**DOI:** 10.1038/s41598-023-46643-6

**Published:** 2023-11-20

**Authors:** Indhumathi. S, J. Christopher Clement

**Affiliations:** School of Electronics Engineering, Vellore Institute of Technology, Vellore, India

**Keywords:** Electrical and electronic engineering, Engineering, Mathematics and computing

## Abstract

Augmented Reality (AR) is applied in almost every field, and a few, but not limited, are engineering, medical, gaming and internet of things. The application of image tracking is inclusive in all these mentioned fields. AR uses image tracking to localize and register the position of the user/AR device for superimposing the virtual image into the real-world. In general terms, tracking the image enhance the users’ experience. However, in the image tracking application, establishing the interface between virtual realm and the physical world has many shortcomings. Many tracking systems are available, but it lacks in robustness and efficiency. The robustness of the tracking algorithm, is the challenging task of implementation. This study aims to enhance the users’ experience in AR by describing an image using Directional Intensified Features with Tertiary Filtering. This way of describing the features improve the robustness, which is desired in the image tracking. A feature descriptor is robust, in the sense that it does not compromise, when the image undergoes various transformations. This article, describes the features based on the Directional Intensification using Tertiary Filtering (DITF). The robustness of the algorithm is improved, because of the inherent design of Tri-ocular, Bi-ocular and Dia-ocular filters that can intensify the features in all required directions. The algorithm’s robustness is verified with respect to various image transformations. The oxford dataset is used for performance analysis and validation. DITF model is designed to achieve the repeatability score of illumination-variation , blur changes and view-point variation, as 100%, 100% and 99% respectively. The comparative analysis has been performed in terms of precision and re-call. DITF outperforms the state-of-the-art descriptors, namely, BEBLID, BOOST, HOG, LBP, BRISK and AKAZE. An Implementation of DITF source code is available in the following GitHub repository: github.com/Johnchristopherclement/Directional-Intensified-Feature-Descriptor.

## Introduction

Each image has its unique characteristics, which can be described by features. Features are the necessary information to identify the image by the machine. The role of a feature descriptor is to extract features and describe the image through them. The effectiveness of feature extraction decides the Robustness of a descriptor. Feature detection and description are the important process of Computer Vision (CV) applications. The fundamental step of CV applications rely on detecting the features for further process. A few recent applications of CV include, Autonomous Vehicles, Factory Automation, Medical Imaging^1–3^ , Human-Computer Interaction, and Augmented Reality (AR).

AR superimpose the virtual kind of digital images over the real world information through AR device. The tracking algorithms are the vital component in this superposition. The accuracy of AR application relies on tracking algorithm, which in turn depend on robust feature description. This idea motivated us to design the light-weight feature descriptor for AR applications. Nowadays, AR provides more impact in our daily life due to the development of CV technology. This development can still be enhanced and implemented in various fields. For example, AR is used in all kinds of field to name a few, Education^4,5^, Medical, Industry, and Entertainment. This AR is an emerging technology, and it demands a lots of development in this field. The overview of the AR generation is as follows.

- The input image is given to feature descriptor for pre-processing, detection and extraction of the features.
- The extracted feature is given to matching technique to match the feature of the test image, with reference image.
- The matched feature is given to pose estimation to create a 3D model/AR output.

The performance of AR model is measured by two parameters: (i) Robustness, and (ii) Efficiency. Robustness is an ability of a descriptor to effectively describe the features even when the image undergoes any transformations. Efficiency is measured in the form of time taken to process the image into an AR. The feature descriptor is robust, if it is

- Compact in size/dimension
- Feature should remain same irrespective of the nature of the image and scenes.
- Invariant to rotation, scale and lighting changes.

Many feature descriptor models are found in the literature, namely, Scale Invariant Feature Transform (SIFT), KAZE, Speeded Up Robust Features (SURF), Binary Robust Invariant Scalable Keypoints (BRISK), Oriented FAST and Rotated BRIEF (ORB), Fast Retina Keypoint (FREAK) and Histogram of Oriented Gradient (HoG). Based on our survey, each model is using different method to detect the features, which leads to achieve a robustness and efficiency.

SIFT uses multiple scale of images and extract the feature of those images using Difference of Gaussian method. Many modified SIFT^6^ has been released recently for the improvement of image matching. The feature detected from SIFT is invariant to size and orientation of the image. Despite, SIFT features, retrieves the image from large set of database, It has limitation such as computation speed.

The descriptor SURF uses Hessian matrix to locate objects / images. It utilizes the box filter in gray scale image for the improvement of computation in image detection and tracking^7^. A new SURF model, involves the multi-modal images to predict the feature matching which leads to achieve the quality image registration^8^. However, feature extraction of SURF is not stable in illumination and rotation variation.

ORB make use of FAST detector for feature extraction as name implies it is faster^9^. The Multi-scale pyramid model of ORB represents a scale invariant property. However, accuracy of this property in ORB is less than SIFT^10,11^. The intensity centroid technique attributes the rotation invariance. ORB provides high immune to Gaussian noise. In outdoor environment, ORB predicts more features than SIFT and SURF but it lacks in robustness.

The FREAK descriptor’s feature selection is based on retinal sampling pattern. In terms of computation speed, FREAK is better than ORB and BRISK and is 29.1 ms for mobile AR tracking^12^. Generally, for the AR augmentation, the computation time should be less than 100 ms. FREAK achieves the speed by reducing the dimension of the descriptor, but at the cost of compromising its tracking accuracy.

kAZE descriptors are used to describe 2D features in a nonlinear scale space by means of nonlinear diffusion filtering. They operates in nonlinear scale space. The Gaussian scale space used in SIFT and SURF does not obey the natural boundaries of object. In contrast, nonlinear scale space preserves the image details as long as it’s necessary. Modified KAZE features are employed to detect the Diabetic Retinopathy (DR) in medical field^13^. This method can detect DR in early stages, with minimum amount of processing time^14^.

HoG^15^ is used in many CV applications starting from pedestrian detection in static image to human detection in motion (video). However, the performance of HoG lacks in light and scale in-variance. Recently, many modified version of HoG have been published in object detection and matching^16^. In Ref.^17^, the authors have proposed the Linear Binary Pattern (LBP) combined with HoG model to improves the accuracy of feature extraction. Therefore, this model is used in early-stage detection of Congenital heart defects to prevent adverse consequences. HoG provides a good image matching, although it has the limitation such as rotation-invariance.

Applications like autonomous vehicle and augmented reality needs high resistance in light and rotation-variation of image matching. Adversarial learning-based feature extraction uses day and night features to improve the image matching in illumination variation^18^. However, it need prior information of scene for reconstruction. The new GAN model has been published to establish the 3D reconstruction without using any prior data^19^. In contrast, it needs more image to train a model and time consuming.

Image matching in AR tracking engages Spherical localization algorithm^20^ to identify the position of stationary users. Spherical localization algorithm adopt the technique of global localization to extract 1000 features for further processing. However, the tracking is consistently poor due to the feature matching. This factor limits the successful tracking, for long period of time. In^21^, the researchers have proposed multi attention model, to improve the feature matching and it enhances the tracking efficiency.

Real-time application of AR tracking requires robust features for effective localization.

In recent years, many feature descriptor models have been published for the improvement of feature matching^22,23^. However, each model satisfies only one property of descriptor as we mentioned in Introduction . Our aim of the proposed model is to fulfil the gap, which are in mismatch and weakness of the descriptor towards various image transformations. To improve the robustness of the feature descriptor, we are implementing the following steps in our proposed work.

In summary, the main contributions of our work with respect to our preliminary study are as follows:

1. The DITF method computes the features of an image using three inherent filters, namely Tri-ocular, Bi-ocular, and Dia-ocular.
2. We introduced a directional intensification algorithm to describe the features in all required directions.
3. The DITF model is made in such a way that it is invariance to affine transformations.
4. The performance of DITF is measured in terms of the repeatability score for the region of interest.

Hence, DITF model implies the enhancement of robustness in AR tracking.

The remainder of this paper is organised as follows: The proposed methodology is given in The proposed model with the significance of the filter design. Results and Discussion investigates and estimates the performance of the feature descriptor. Finally Conclusion and Future Scope is mentioned in Conclusion and Future Scope.

**Figure 1:**
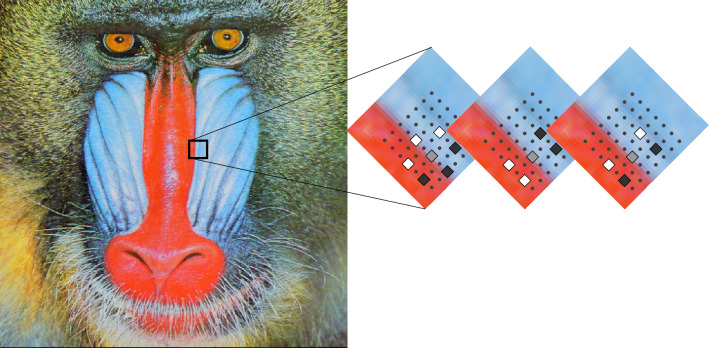
Tri-ocular ,Bi-ocular and Dia-ocular filter applied to sample image plane.

**Figure 2:**
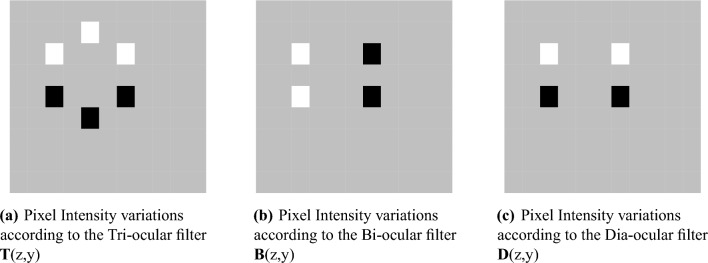
Filter design used in proposed model.

## The proposed model

In this section we will discuss the implementation of DITF Model. We propose a feature extraction model for feature matching that is calculated based on direction of pixels. Our method is different from existing descriptor that it make use of three filters for computing the direction of pixels. The performance result has been analysed using repeatability score. Here, we describe our feature extraction for the image size of M*N and the image matrix is represented as *A* in Eq. (1). From the image matrix we segmented the sample pane as $A_{ij}$. The sample plane size should be same as filter size. We design a filter in such a way that it has to analyse the three direction of pixels for the feature validation. The size of the filter is taken as *k* and *l*. Apply each filter to the sample plane as illustrated in Figs. 1 and 2. Each filter consist of the values namely,-1,1 and 0 in various spatial location as per the requirement. The filters *T*(*z*, *y*), *B*(*z*, *y*) and *D*(*z*, *y*) respectively are designed based on Eqs. (2), (3) and (4).

Tri-ocular filter act as a parallel filter to measure the parallel plane of the image. Bi-ocular filter obtain the perpendicular values. Dia-ocular filter evaluates the plane that connect non-adjacent vertices of the image. $A_{ij}$ is transformed to vector as given in Eq. (5).

**Figure 3:**
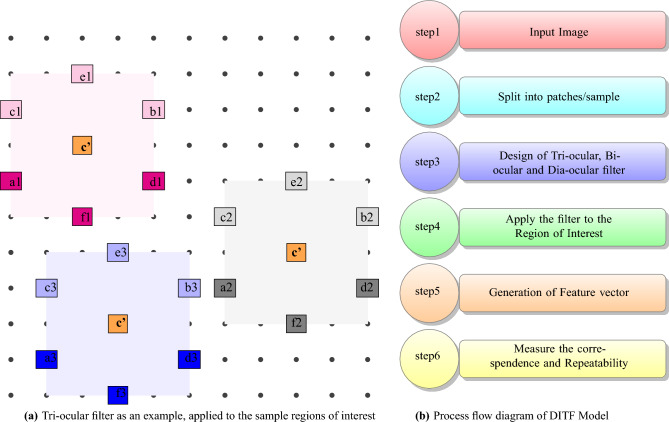
Tri-ocular filter operation and DITF flow diagram.

The filter computes the directional intensification with the help of Eqs. (6) and (7) to provides the corner and edge details of the sample image matrix $A_{ij}$. The algorithm used to acquire the feature vector of the whole image plane is shown in 1.

The details in the given 2 dimensional image plane *A* might be of variations in the intensity in any particular direction. For the better description, these features, must be highlighted with their intensities along with the direction they point to. Let $\theta$ denote an angle from $0^\circ$ to $\Phi ^\circ$ and is divided linearly into *R* number of points equally, with the step size $\Delta _\theta$, such that $\Delta _\theta = \dfrac{\Phi ^o}{R}$. Let the incremental values of $\theta$ be $\theta _0, \theta _{1}, \cdots , \theta _{R}$. With $G_{ij}$, $H_{ij}$ and $\theta$, the feature describing algorithm go on as below. *I* is plotted as per algorithm 1 to evaluate the feature distribution in image. With respect to *I* we need to find the orientation of $\theta$ along with the variation in intensity. Each $\theta$ developed from the algorithm 2 is the feature vector of the descriptor. Every region of interest in image generates one feature vector. The sample plane, filter size and classes decides the dimension of the feature descriptor. The dimension of the DITF descriptor is ((k*l) *R). The DITF model flow diagram is given in Fig. 3.

The entire 2 dimensional gray scale image can be written in matrix form as

1 $$\begin{aligned} {\textbf {A}} = \left[ \begin{array}{cccc} a_{11} &{} a_{12} &{}\cdots &{} a_{1N} \\ a_{21} &{} a_{22} &{}\cdots &{} a_{2N} \\ \vdots &{} \vdots &{} \vdots &{} \vdots \\ a_{M1} &{} a_{M2} &{} \cdots &{} a_{MN} \\ \end{array} \right] \end{aligned}$$

where *M* and *N* denote the size of the matrix *A*, *M* is number of rows and *N* is number of columns, respectively.

In order to do the filtering, we define three filters, namely, ’Tri-ocular’, ${\textbf {T}}$, ’Dia-ocular’, ${\textbf {D}}$, and ’Bi-ocular’, ${\textbf {B}}$.

For the defined $u = \lfloor l/2\rfloor +\mod (l,2)$, where$\lfloor \cdot \rfloor$ is the floor operation, the elements of the ’Tri-ocular’ filter ${\textbf {T}}(z,y) \;;\; z = 1,2,\cdots , k\; ; \; y = 1,2,\cdots , l$, whose values are filled with 0, -1 and 1 as follows,

2 $$\begin{aligned} {\textbf {T}}(z,y) = \left\{ \begin{array}{ccccc} -1 &{}; &{} z = 1,u-1,u-1 &{}; &{} y = u,1,l \\ 1 &{}; &{} z = u+2,u+2,2u &{}; &{}y = 1,l,u \\ 0 &{}; &{} \text {else} \end{array} \right. \end{aligned}$$

Similarly, the ’Bi-ocular’ and ’Dia-ocular’ filters are defined, respectively, as

3 $$\begin{aligned}{} & {} {\textbf {B}}(z,y) = \left\{ \begin{array}{ccccc} -1 &{}; &{} z = u-1,u+2 &{}; &{} y = 1,1 \\ 1 &{}; &{} z = u-1,u+2, &{}; &{} y = l,l \\ 0 &{}; &{} \text {else} \end{array} \right. \end{aligned}$$

4 $$\begin{aligned}{} & {} {\textbf {D}}(z,y) = \left\{ \begin{array}{ccccc} -1 &{}; &{} z = u-1,u-1 &{}; &{} y = 1,l \\ 1&{}; &{} z = u+2,u+2 &{}; &{} y = 1,l \\ 0 &{}; &{} \text {else} \end{array} \right. \end{aligned}$$

From the matrix ${\textbf {A}}$, a segment array ${\textbf {A}}_{{\textbf {ij}}}$ for the varying values of $i = 1,2,\cdots , M-\left( \dfrac{k}{2} + 1\right)$ and $j = 1,2,\cdots , N-\left( l+1\right)$, where $k\ge 6 \; ; \mod \left( k,2\right) = 0$ and $l\ge 5; \mod \left( l.2\right) = 1$, can be sliced for the forthcoming operation.

For the operational convenience, the sliced array ${\textbf {A}}_{{\textbf {ij}}}$ is transformed into ${\textbf {a}}$ as

5 $$\begin{aligned} {\textbf {a}}_{(p-q)l+q} = \left[ {\textbf {A}}_{{\textbf {ij}}} \left( p,q\right) ; \; \forall \; p = 1,2,\cdots , k; \forall \; q = 1,2,\cdots , l \right] \end{aligned}$$

Given the filters, and for the fixed *i* and *j*, the magnitude metric is given by

6 $$\begin{aligned} G_{ij} = \sqrt{ \left( {\textbf {a}}\cdot {\textbf {b}}\right) ^2 + \left( {\textbf {a}}\cdot {\textbf {t}}\right) ^2 +\left( {\textbf {a}}\cdot {\textbf {d}}\right) ^2} \end{aligned}$$

where ${\textbf {b}}$, ${\textbf {t}}$ and ${\textbf {d}}$ are the similar transformation of filters ${\textbf {T}}$, ${\textbf {B}}$ and ${\textbf {D}}$ as mentioned in (5).

Similarly, the angles for each sample of *i* and *j* is computed as

7 $$\begin{aligned} H_{ij} = \arctan \left( \dfrac{{\textbf {a }}\cdot {\textbf {d}}}{{\textbf {a}}\cdot {\textbf {b}}}\right) + \arctan \left( \dfrac{{\textbf {a}}\cdot {\textbf {d}}}{{\textbf {a}}\cdot {\textbf {t}}}\right) \end{aligned}$$

**Figure:**
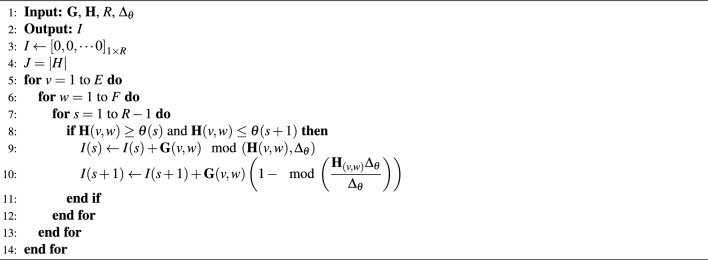
Algorithm 1 Compute gradient aggregation.

**Figure 4:**
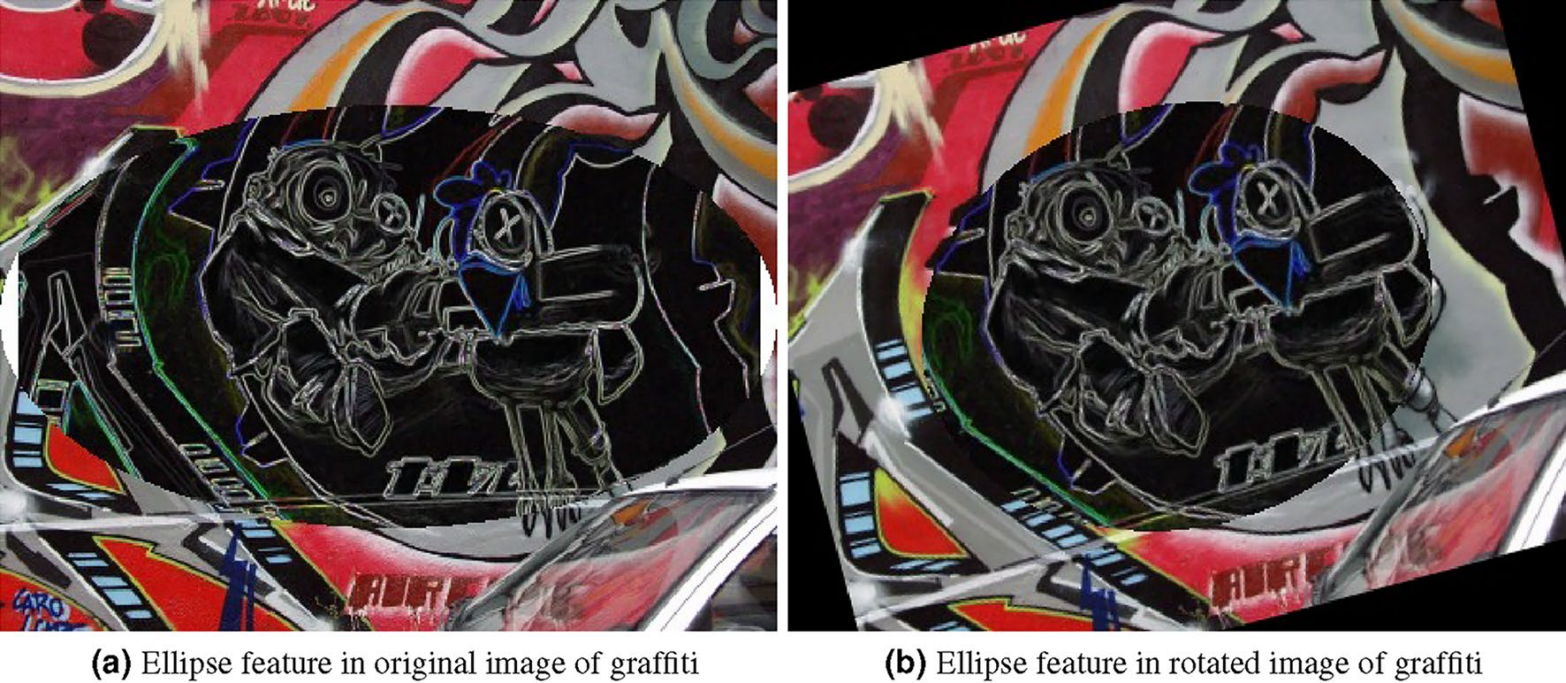
Region of interest feature extraction for graffiti image.

**Figure 5:**
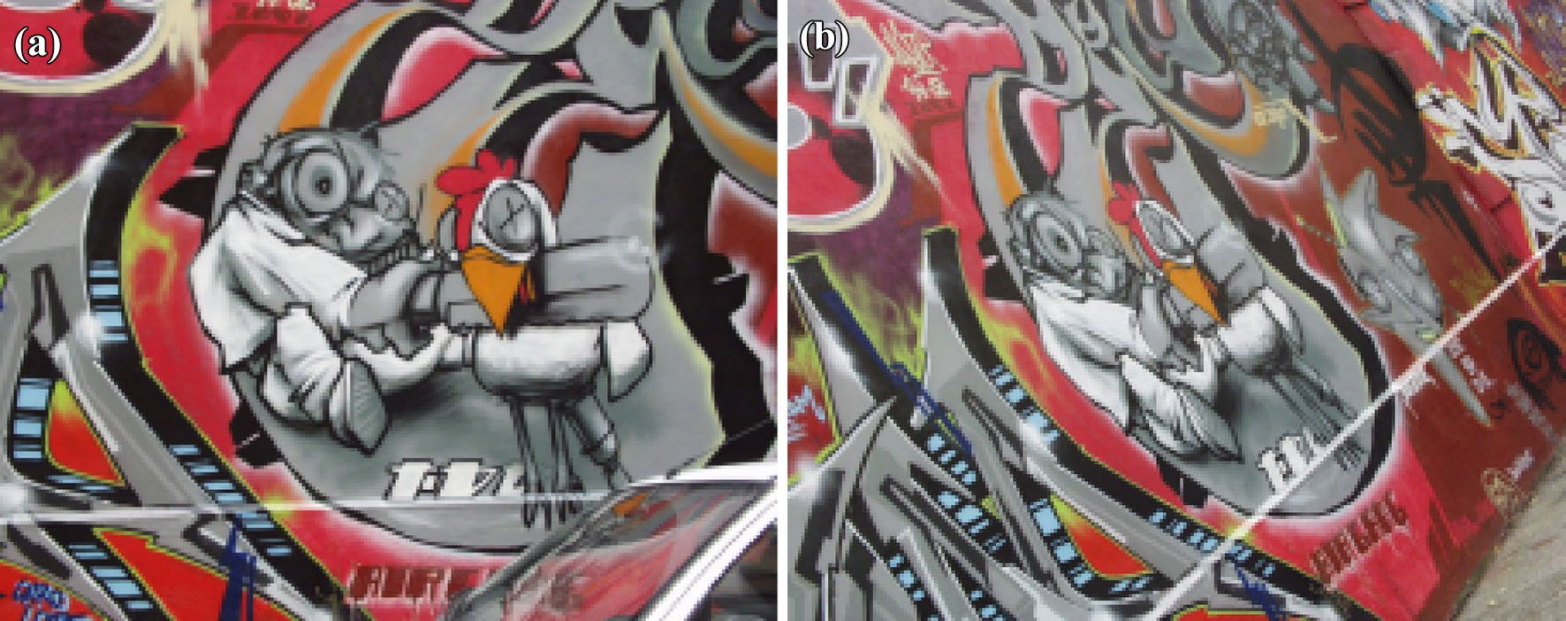
(**a**) original graffiti image (**b**) viewpoint changed by $40^\circ$.

**Figure 6:**
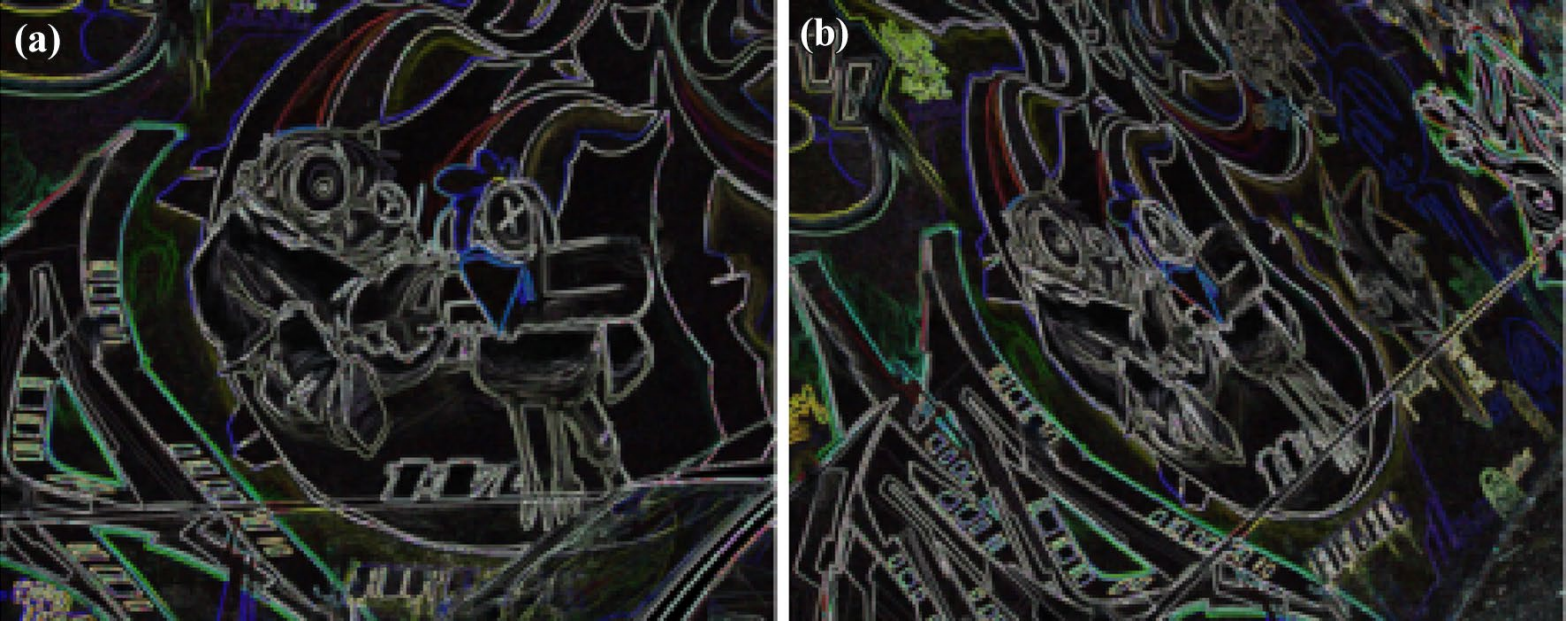
(**a**) Original graffiti image feature (**b**) Feature of viewpoint variation by $40^\circ$.

**Figure 7:**
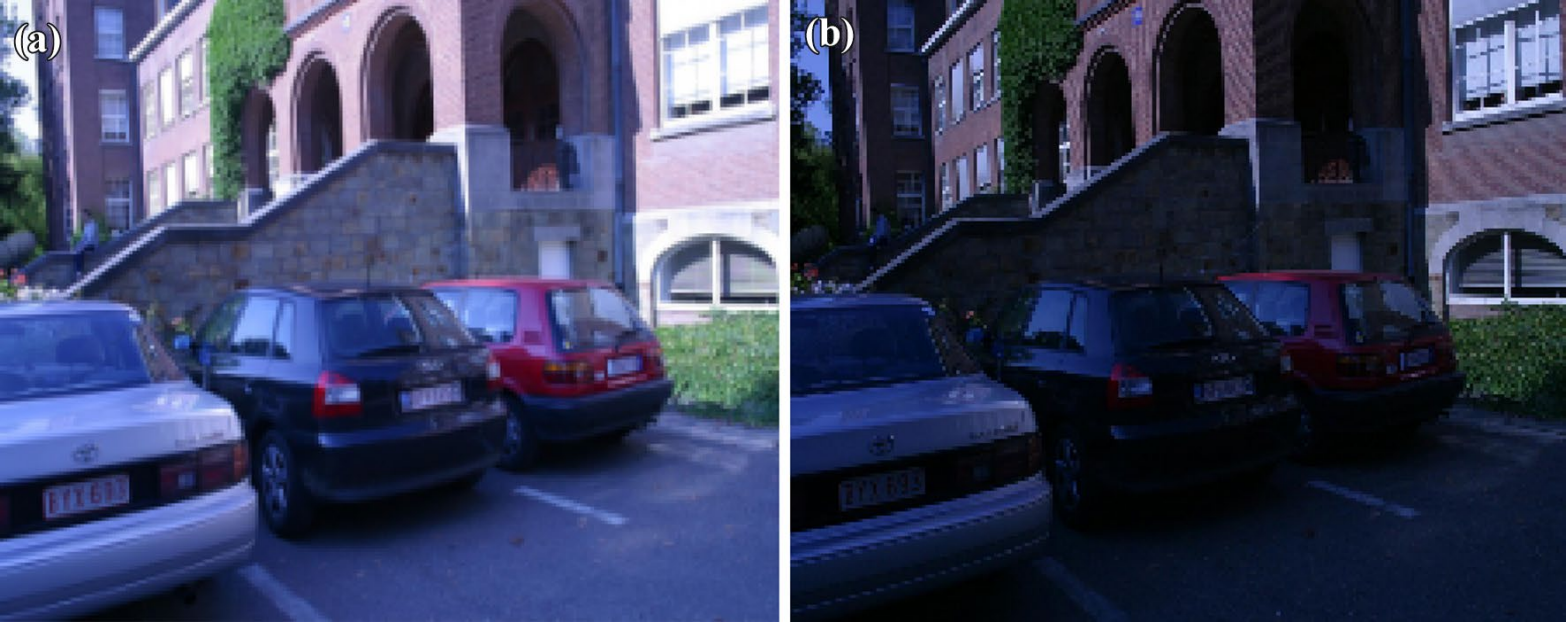
(**a**) Original car image (**b**) Light intensity variation of car image.

**Figure 8:**
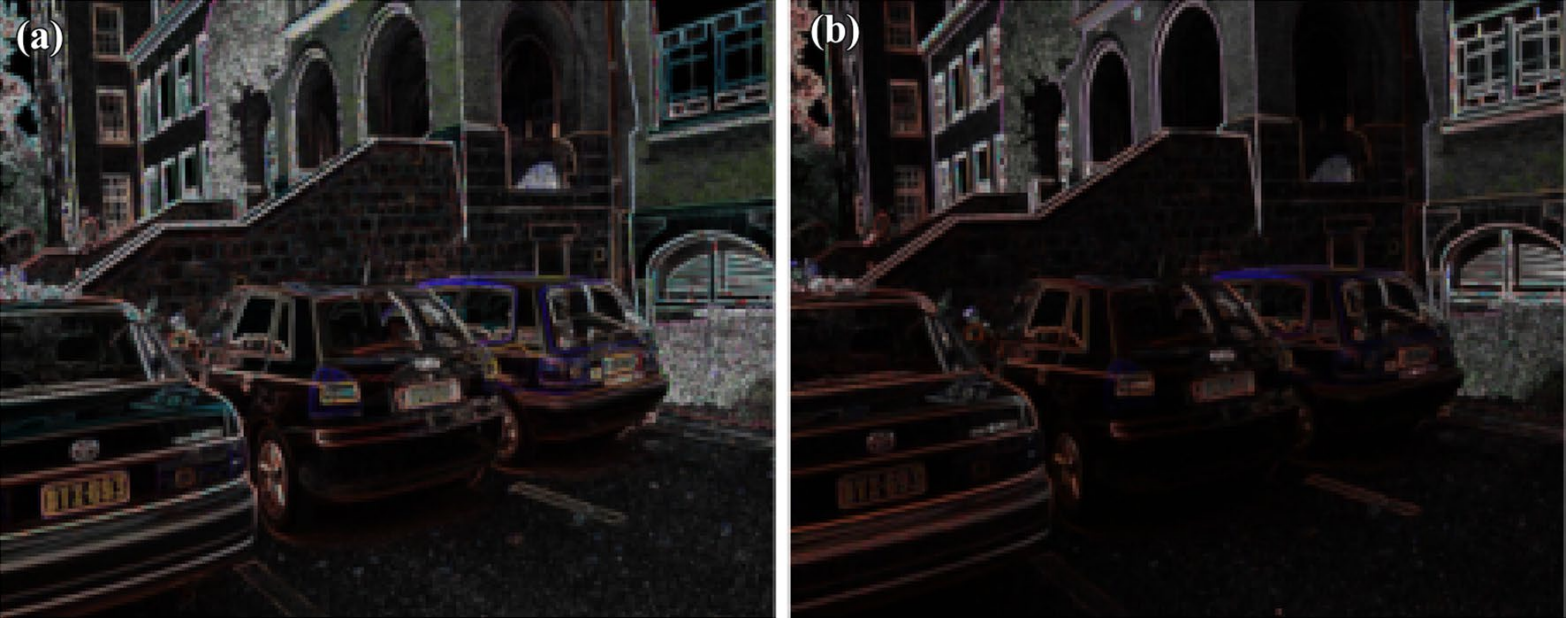
(**a**) Feature extraction of car image (**b**) Feature extraction of Light intensity variation.

**Figure 9:**
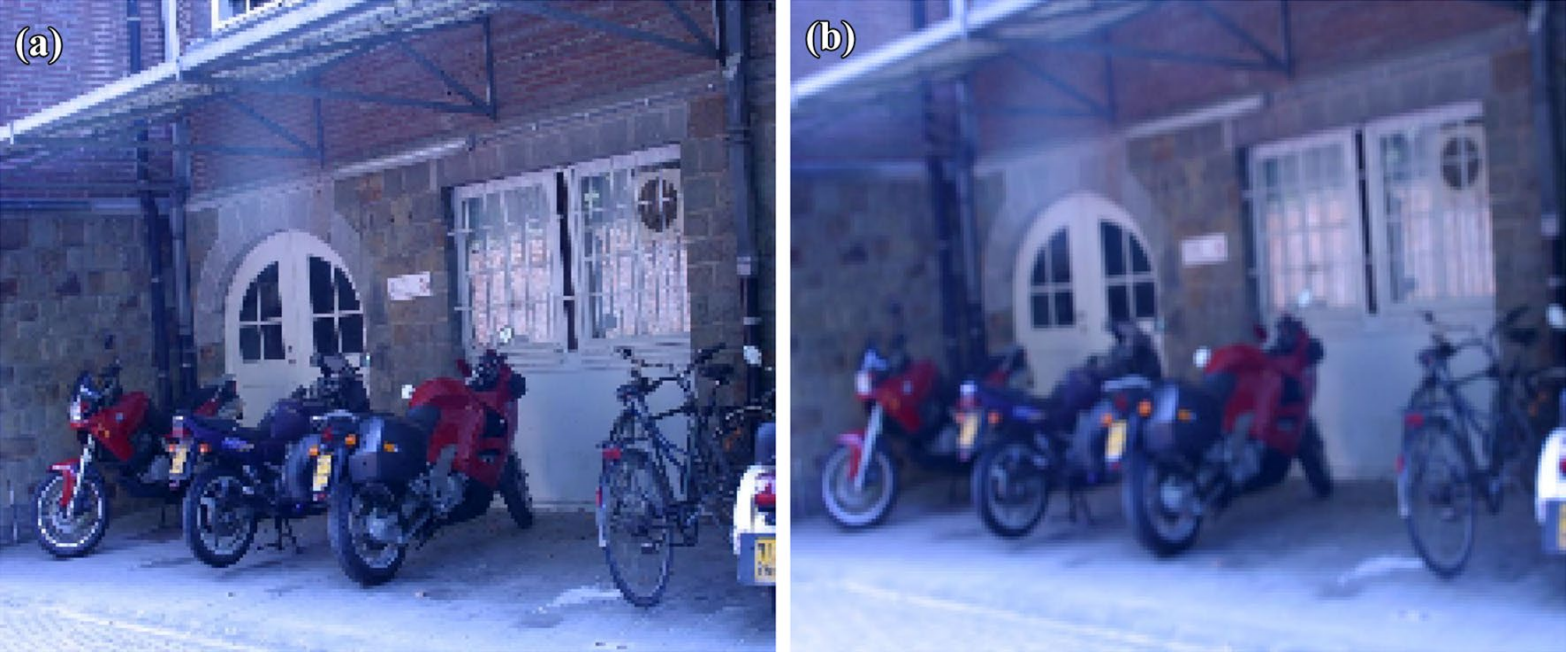
(**a**) Original bike image (**b**) Blur variation of bike image.

**Figure 10:**
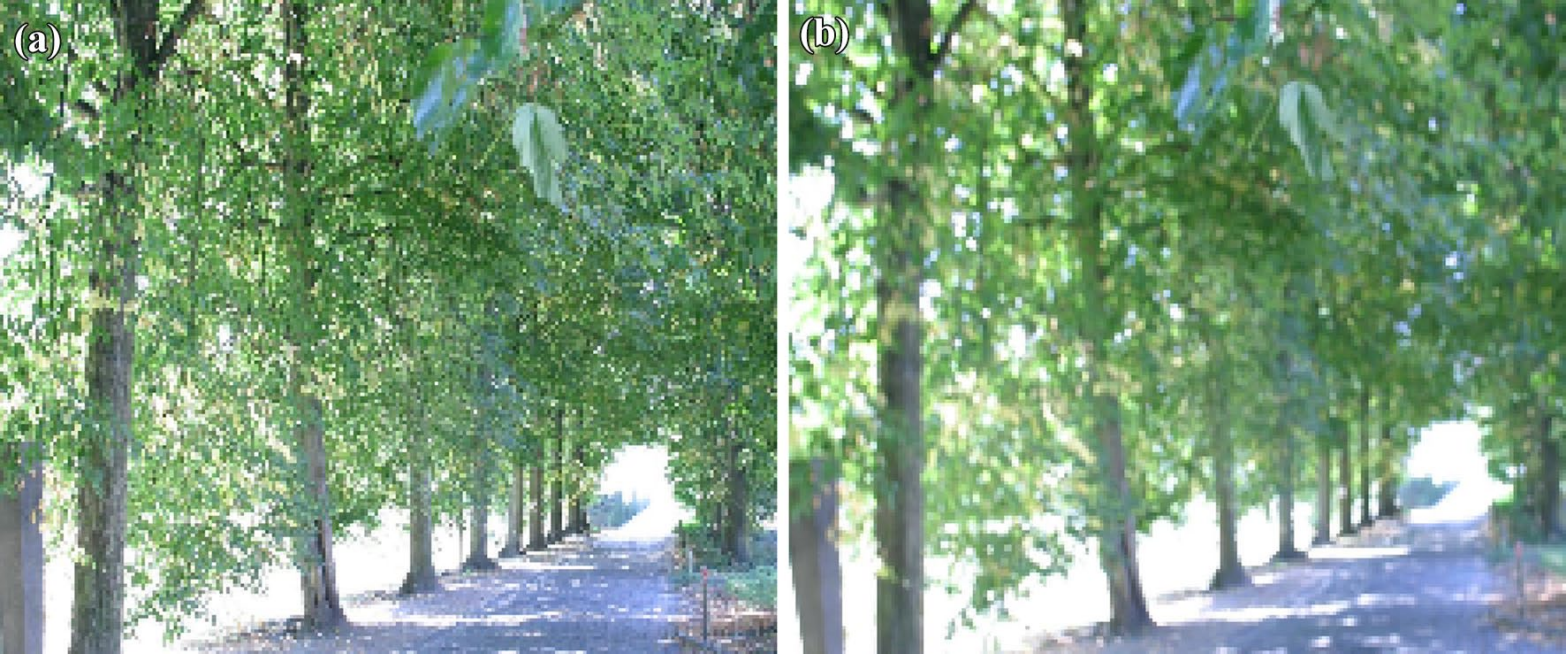
(**a**) Original tree image (**b**) Blur variation of tree image.

**Figure 11:**
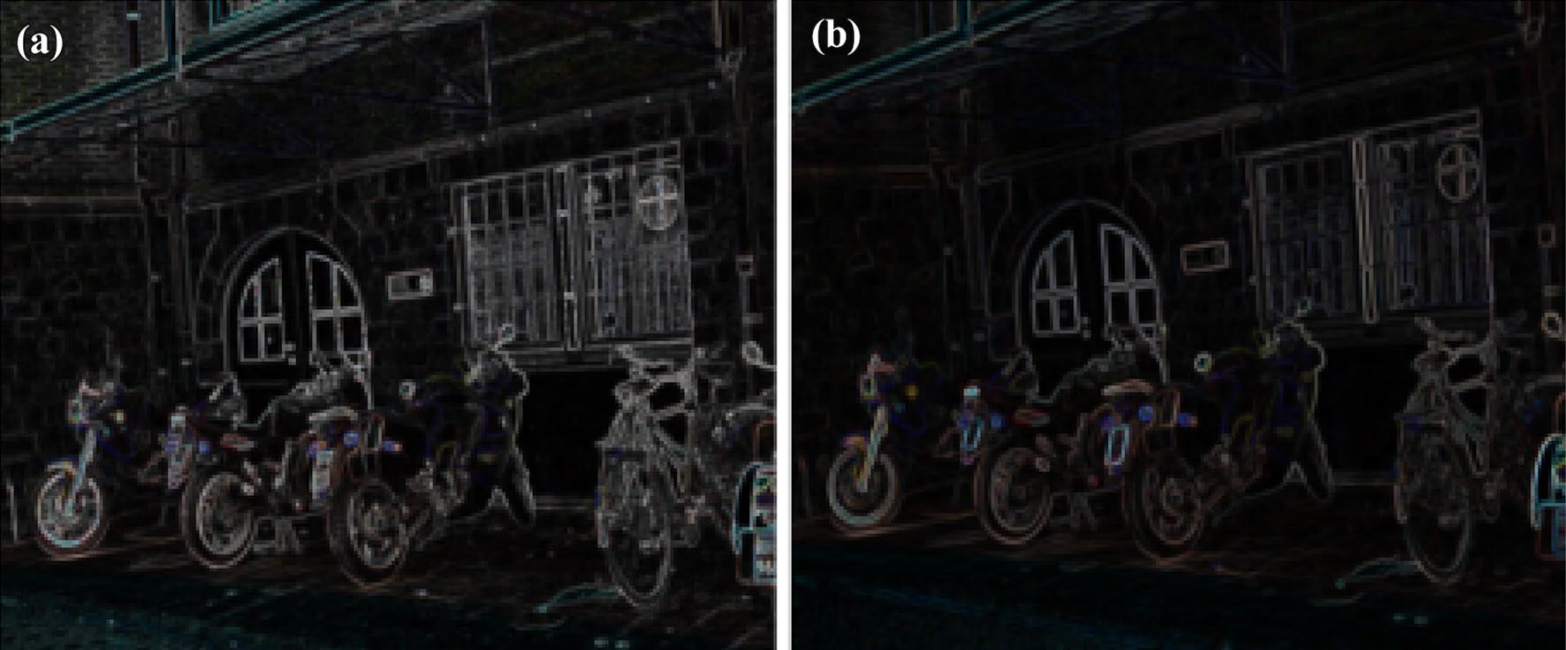
(**a**) Feature extraction of bike image (**b**) Feature extraction of blur variation.

### Input image transformation

The input is taken as a mandrill image and the robustness of DITF model is tested using Oxford Dataset. For further references of dataset you can use the following link https://www.robots.ox.ac.uk/. It has five types of transformation (Blur, View-point, zoom with rotation, Light, and JPEG Compression). As per our analysis, we have included three transformations with different types of images. The reason is, these three transformations are occurred frequently in AR Tracking. Each transformation has six variations of images. We have analyzed all six images and we have included significant image for validation. Mandrill image is taken from USC university of southern California, the link is attached here for the reference of database image http://sipi.usc.edu/database/. The effective feature descriptor^12^ should be robust to scale, rotation and lighting changes of the image. We applied affine transformation to the original image using homography matrix. Oxford Dataset used in our algorithm is as follows:

- View-point variation
- Blur changes
- Illumination variation.

All the images used in our analysis is medium resolution approximately 800*640 pixels. All affine transformation image features are extracted using DITF. The relevant figure is illustrated in section Input Image Transformation

#### Homography

Homography matrix establish a relation between two images of same planar with different transformation. The homography between reference and test image is computed in two steps:

- Manually, select four correspondence point between two images.
- Mapping between these two projection are represented by 3*3 homogeneous matrix which is shown in equation (8).

The Eq. (8) is used to understand the homography principle. The relation of h-matrix between two images are given. The reference image plane co-ordinates are (x,y,1), we are getting warped /transformed image as ($x{'}$,$y{'}$,1) with the help of h-matrix. Hence, the affine co-ordinates of any point can be computed in Homography matrix. DITF make use of homography matrix to measure the transformation and establish the robustness of our feature descriptor model. The attribute of homography is independent of scene/ environment^24^. Hence, It is very much useful in AR.

8 $$\begin{aligned} \begin{bmatrix} x'\\ y'\\ 1 \end{bmatrix}= \begin{bmatrix} h_{11} &{} h_{12}&{}h_{13} \\ h_{21} &{} h_{22} &{}h_{23}\\ h_{31}&{}h_{32}&{}h_{33} \end{bmatrix} \begin{bmatrix} x\\ y\\ 1 \end{bmatrix} \end{aligned}$$

**Figure 12:**
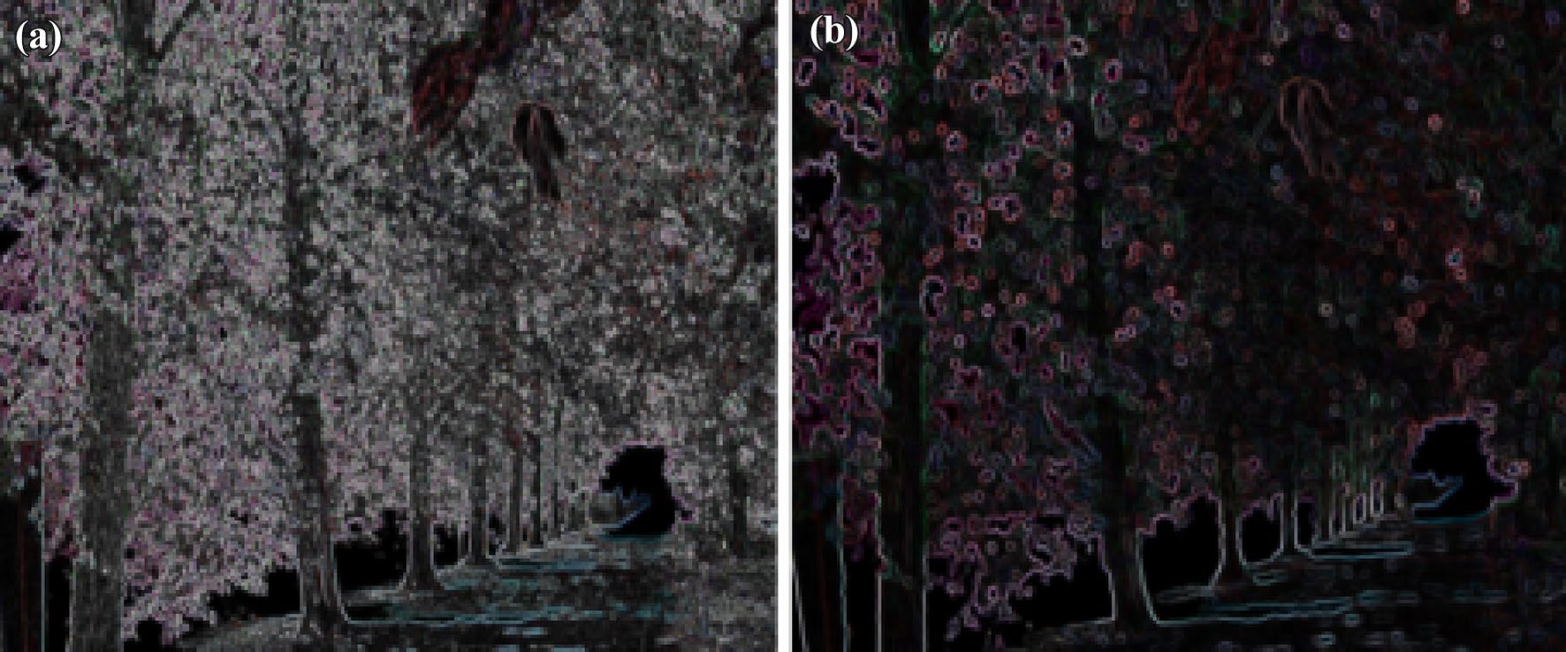
(**a**) Feature extraction of tree image (**b**) Feature extraction of blur variation.

**Figure 13:**
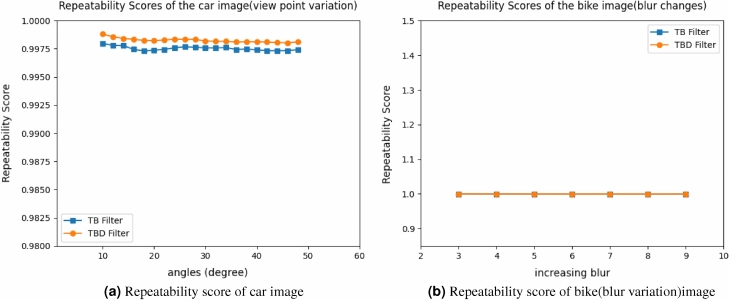
Repeatability score of car and bike image.

**Figure 14:**
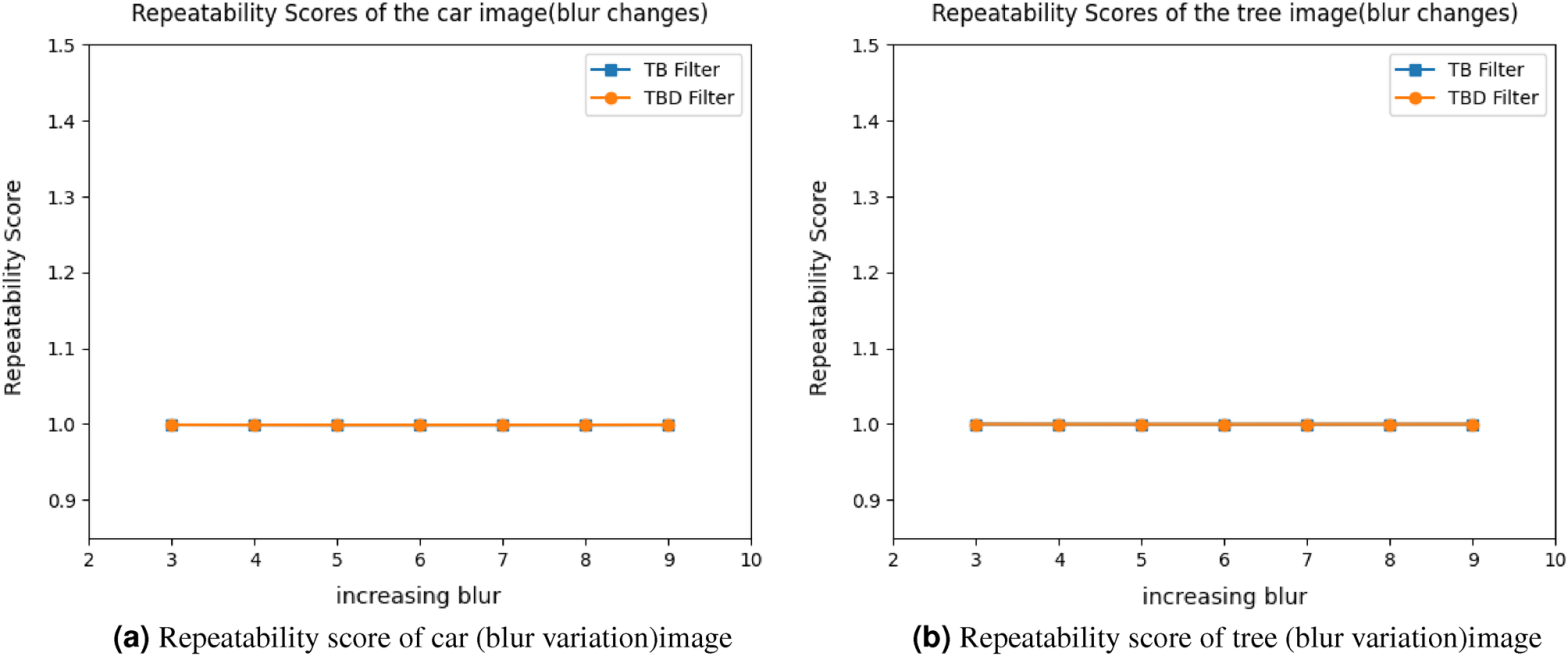
Repeatability score of blur image for car and tree image.

## Results and discussion

This section discuss the simulation results and analysis of the feature descriptor:

The comparison analysis of two combination of filter was designed namely,

- Tri-ocular and Bi-ocular filters (TB)
- Tri-ocular, Bi-ocular and Dia-ocular filters (TBD).

1. We plotted an ellipse region on test image that made use of homography matrix, features are extracted in region of interest for correspondence matching.
2. Repeatability score is obtained with respect to the feature matching between two images.
3. The performance measurement of our DITF model is analyzed and compared with state-of-the-art descriptor.

The simulation results of our model, is implemented by anaconda3 python 3.9.12 in Jupyter notebook with CPU i7-11390H @3.40GHz. This section includes the discussion of "Correspondence and repeatability measurements" and "Results and discussion".

**Figure 15:**
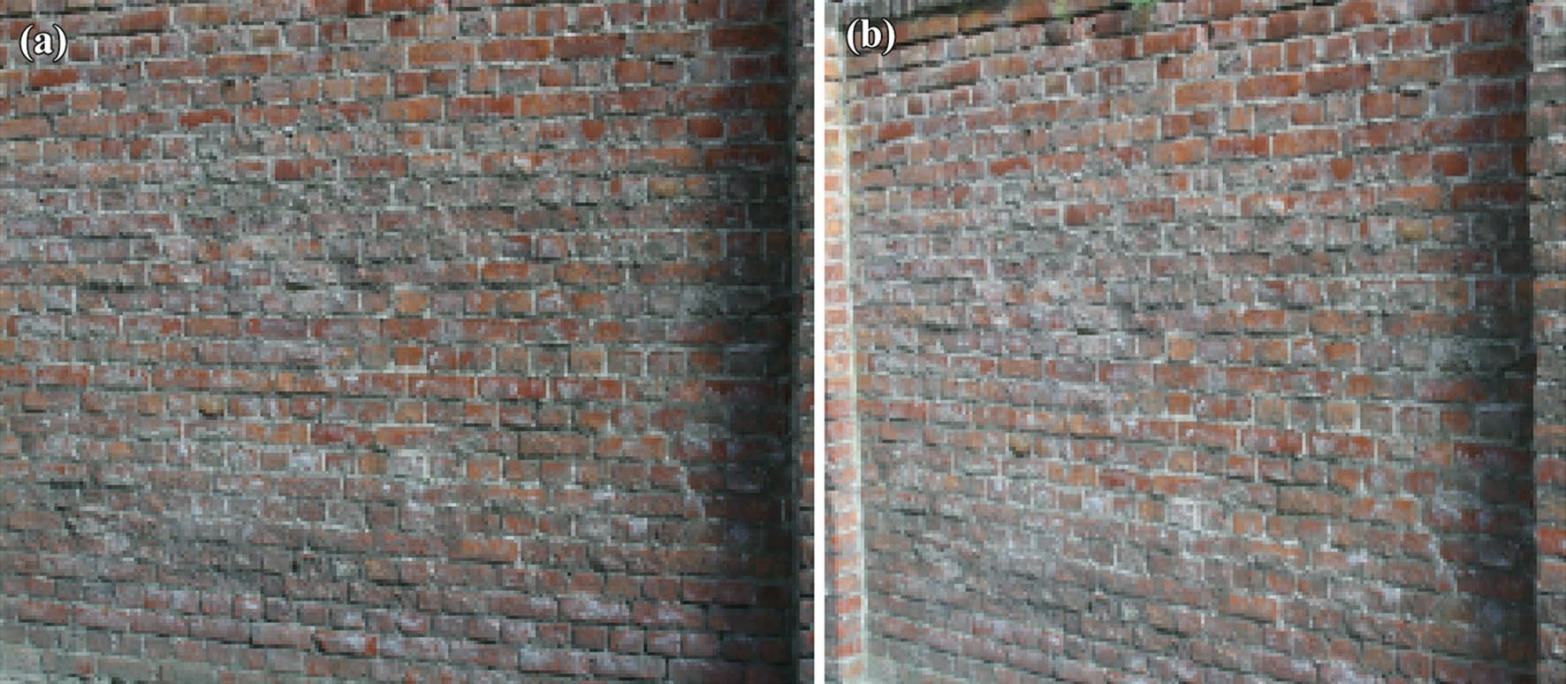
(**a**) Original wall image (**b**) View-point variation of wall image.

**Figure 16:**
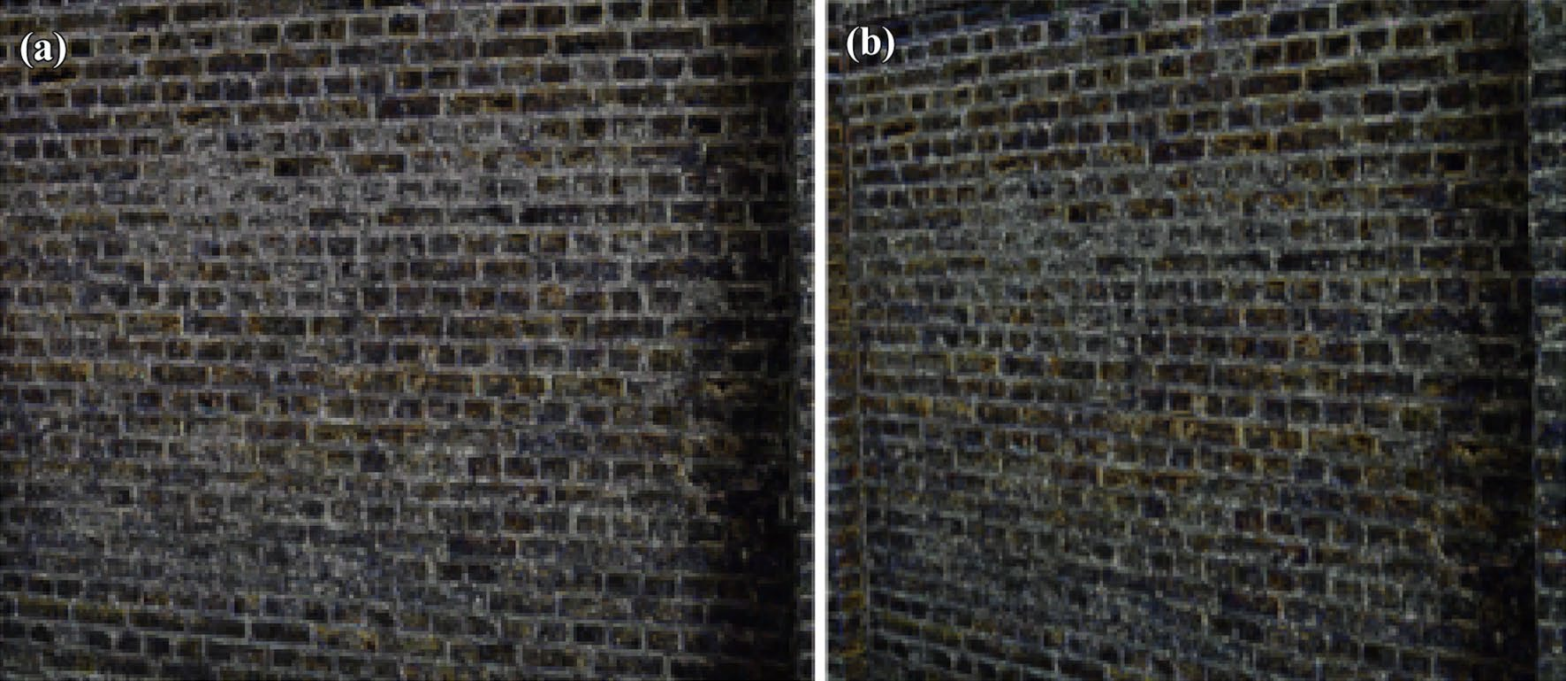
(**a**) Feature extraction of wall image (**b**) Feature extraction of View-point variation.

### Correspondence and repeatability measurements

Region is a set of pixel with similar kind of properties. For accurate interpretation of image we plotted ellipse pair in reference and test image. Homography matrix of graffiti are given in Eq. (11). Feature extraction in ellipse region make use of DITF model. Figure 4 shows the graffiti view-point varaition of angle $30^\circ$. All the features are overlapped and matched between two images in the overlap region. This shows the accuracy of our proposed model. The overlap error($\epsilon$) is measured, to find the true correspondence in image. From Eq. (10) ’H’ is homography, ’A’ is the ellipse region of ground truth image and ’B’ is the ellipse region of transformed image. The overlap error should not be greater than 50% for effective feature descriptor.

**Figure:**
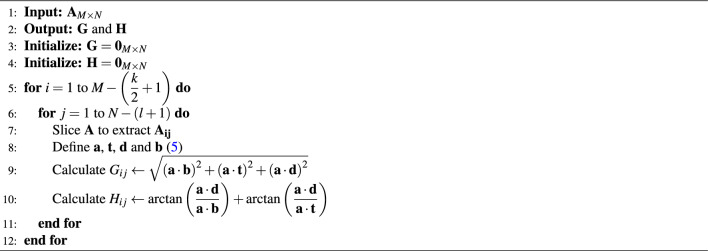
Algorithm 2 Generating feature vector using DITF feature descriptor.

The key evaluation of image matching is Repeatability. The feature between reference image and query image need to be repeated or same for image matching. Matching is obtained from the overlapping between two image features. This provides the correspondence of the image. In accordance with correspondence value, we can calculate the Repeatability. Repeatability score shows the feature descriptor ability to measure the same feature points irrespective of imaging conditions with *N* number of times. If the repeatability score is high, then it has maximum overlapping between images. Repeatability score is measured by the ratio of number of correspondence to total number of features given in Eq. (9).

9 $$\begin{aligned} \text {Repeatability}=\dfrac{\text {Correspondences}}{\text {Total features}} \end{aligned}$$

Correspondence matching is an essential factor in CV applications namely, retrieving camera pose, 3D reconstruction, image classification, image stitching, tracking and image registration. Therefore, DITF model evaluates the correspondence, which is necessary for AR Tracking.

10 $$\begin{aligned} \epsilon =1-\dfrac{A\cap (H^TBH)}{A\cup (H^TBH)} \end{aligned}$$

**Figure 17:**
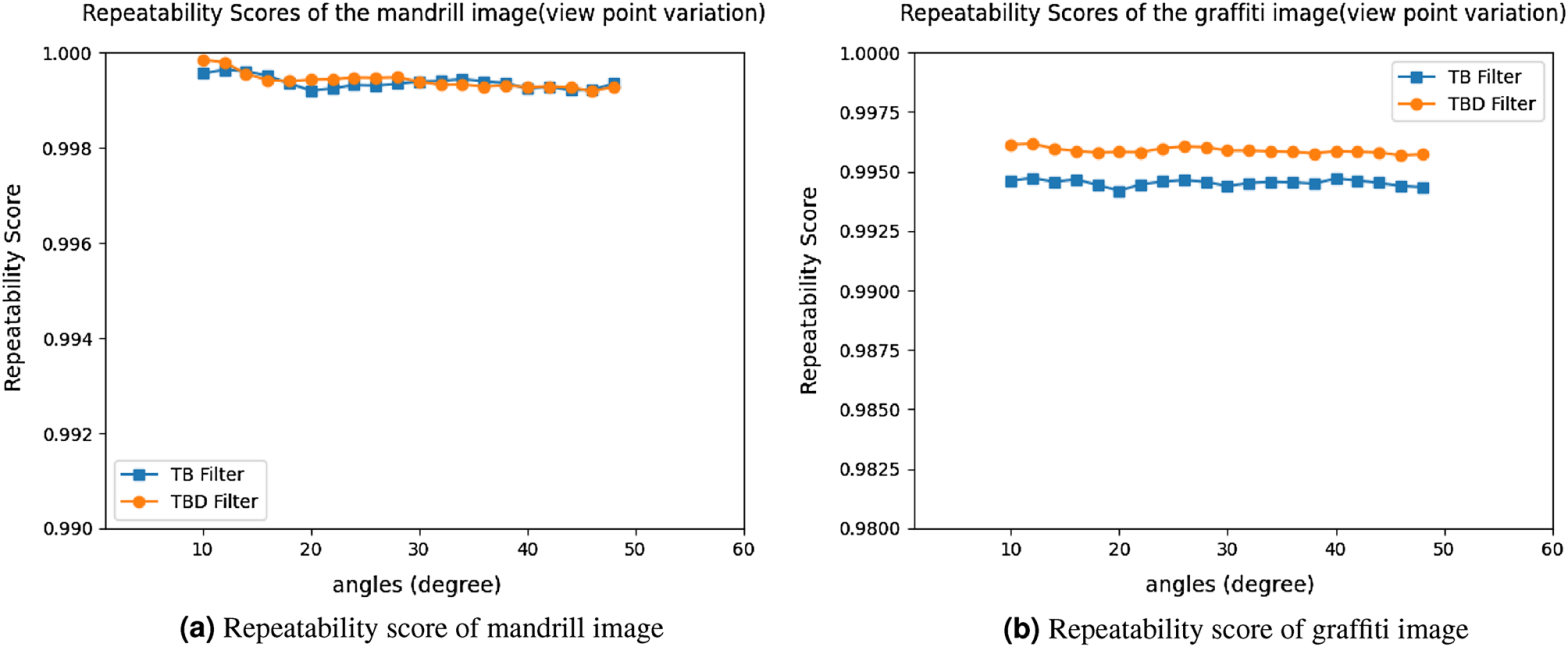
Repeatability score of mandrill and graffiti image.

### Simulation results and discussion

This section analysis the repeatability score of view-point variation, blur image and illumination variation using Oxford Dataset. The feature vector is measured based on two ways as mentioned in section Results and Discussion. (1) The first measurement of feature vector utilizing both Tri-ocular and Bi-ocular filter (TB). (2) The second way of feature vector is measured by combining Tri-ocular, Bi-ocular and Dia-ocular filter (TBD). For ideal case, the repeatability score should be 100%. Indeed, the score is decreases due to the mismatch arises in feature matching between the reference and query image.

**Figure 18:**
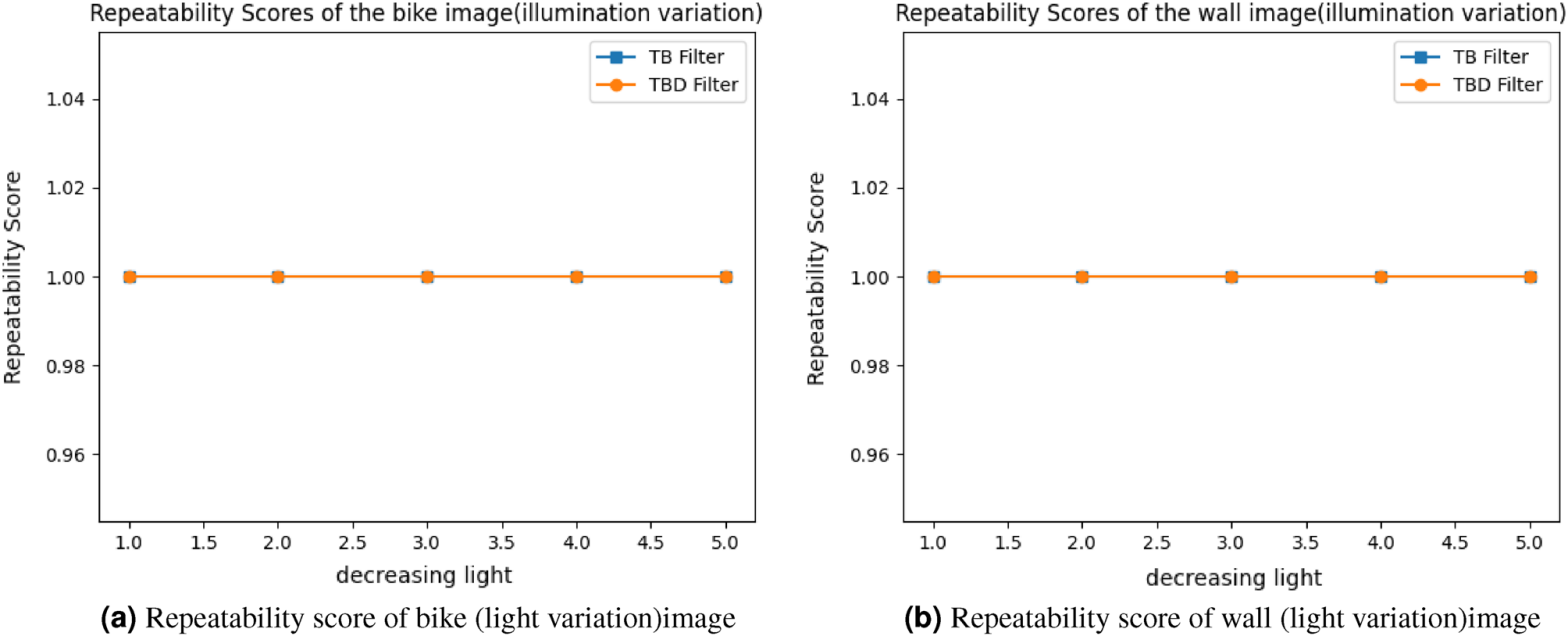
Repeatability score of light variation.

11 $$\begin{aligned} \begin{bmatrix} x'\\ y'\\ 1 \end{bmatrix}= \begin{bmatrix} 7.6285898e-01 &{} -2.9922929e-01&{}2.2567123e+02 \\ 3.3443473e-01 &{} 1.0143901e+00 &{}-7.6999973e+01\\ 3.4663091e-04 &{}-1.4364524e-05 &{}1.0000000e+00 \end{bmatrix} \begin{bmatrix} x\\ y\\ 1 \end{bmatrix} \end{aligned}$$

Figure 5 shows the standard reference image and test image of view-point variation. The DITF features of reference image is compared with $40^\circ$ variation, is shown in Fig. 6. From the output, maximum number of features are extracted in both the images, which shows the retrieval of the shape of the feature in view-point variation. Figure 7 displays the light intensity variation of car image. Light intensity is decreased in the car image and tested the illumination-variation of DITF model. Figure 8 illustrates the DITF features of light variation. Usually, feature descriptor struggles to extract the feature in light variation. However, our model owing three filters, which improves the feature extraction in light illumination. The Figs. 9 and 10 are the two examples of blur image. Two different types of images are included for the blur variation analysis. Bike image has more edges and tree image consist of smooth surface with repetition of the scenes. The blur variation occurs due to the relative motion between scene and camera. The Figs. 11 and 12 shows the feature extraction of blur image. Even though, both the images are in different in nature our model predicts the feature correctly. This is concluded from our visual feature representation of image and also we validated this result by repeatability score of DITF model which is illustrated in Figs. 13b and 14b. Figure 15 is another example of view-point variation. It contains more edges with homogeneous region and the output is shown in Fig. 16. Almost, all the features are extracted and replicated between the reference and query image. Result proves the robustness of the DITF is high in three transformations with different images.

#### View-point variation

The mandrill image simulation curve is plotted between Repeatability score and angle. The repeatability score of TB filter is 99.5% and TBD filter achieves the score of 99.75%. Figure 17a shows the repeatability curve of mandrill image for the view-point variation. This result shows TBD achieves better result than TB filter. TBD extract the hard features in the image which improves the score of TBD than TB filter. The Fig. 17b shows the result of graffiti view-point variation. Images are rotated and tested from $10^\circ$ to $60^\circ$ variation. TBD filter obtain the score of 99.6% and TB filter attains exactly 99.5%. Figure 13a shows repeatability score of car image. The car image is taken as another example to analyse the view-point variation and score is 100% for TBD filter. TB filter obtain the score of 99.75%. The reason for variation in score is, due to the nature of the image. The graffiti and mandrill are similar kind of hard structures so it almost scored the same value. Car image is smooth image which scored high value. However, the score variation is very minimum and it’s in acceptable range. It proves the stability of DITF model in view-point variation. Comparison of two measurement in view-point variation shows TBD filter is better than TB filters.

#### Light variation

This section discuss the simulation result of light variation. The wall and bike image is taken as an example to simulate the score of light transformation in Fig. 18. Gamma value is used to vary the light intensity in the image. The curve is plotted between repeatability and light intensity. TBD and TB filter obtain the 100% score for illumination variation in both the images. Although, the light intensity is decreased DITF matched all the features between reference and test image. It proves our descriptor is more robust in light invariance.

#### Blur Image

For the blur variation three different input image is taken for measurement. The bike, car and tree image. Figure 13b illustrate the score of bike image for TBD and TB filter measurement. It achieves 100% score. Similarly, car and tree image also obtain the 100% repeatability score and it is shown in Fig. 14. This proves our model outperforms the state-of-the-art descriptors in compression.

From the results, we can conclude the measurement of TB and TBD for view-point variation, blurred image and lighting variation. Results of View-point variation shows TBD is better than TB filter. However, blur changes and light variation obtain 100% score in both the filter including different images. This results revealed TBD is recorded high value than TB filter. It proves our DITF model repeatability score is above 99% for all the above mentioned transformations. Evaluation of our model proves our result achieves high accuracy. The computation complexity of DITF model measured, based on the arithmetic operation involved in the feature extraction process. The number of multiplications needed to compute $G_{ij}$ in Eq. (6) is $6k-8+3$ and number of additions needed is $6l-8$.

Assuming that $\arctan$ values are available as a look up table and the above calculated values are stored in a memory (so that can be reused), the number of multiplications needed to calculate $H_{ij}$ in Eq. (7) is 2 and number of additions needed is 1. As the needed computations are very less for $H_{ij}$, it can be ignored. Therefore, when the entire image is considered, the total number of additions and multiplications required, while ignoring the least contributions, are $\left( 6K-3\right) \cdot \left( l-N\right) \cdot \left( \dfrac{k}{2}-M\right)$ and $\left( 6l-7\right) \cdot \left( l-N\right) \cdot \left( \dfrac{k}{2}-M\right)$, while ignoring the contributions of parameters of least significant values. It is to be noted that the required number of both multiplications and additions are of the order of ${\mathcal {O}}(M N)$.

The system that we use is capable of executing 5000 M floating point operations (FLOPS) per second. So, if the image size is $M = N = 512$ and if $k = 6,l = 5$, then 8516079 multiplications (FLOPS) and 5935449 additions are required. Therefore, the time needed to complete the task is 0.3s. This proves DITF model has good computation.

**Table 1:** The mean average precision and mean average recall—A comparison of proposed algorithm with other algorithms.

Descriptor	Mandrill		Graffiti		Car		Bike		Tree		Wall	
	mAP	mAR	mAP	mAR	mAP	mAR	mAP	mAR	mAP	mAR	mAP	mAR
BRIEF-256	0.5968	0.4068	0.6157	0.3510	0.6084	0.4171	0.6138	0.4281	0.5967	0.4117	0.5840	0.3763
ORB-256	0.6096	0.4193	0.6735	0.4273	0.6084	0.4126	0.6516	0.4257	0.5936	0.4093	0.5790	0.4355
BRISK-512	0.6491	0.4901	0.6898	0.4742	0.5806	0.4490	0.6450	0.5189	0.5908	0.5210	0.6842	0.5166
FREAK-512	0.4138	0.2537	0.4118	0.2030	0.3868	0.2677	0.4445	0.2194	0.3805	0.2819	0.4851	0.2836
AKAZE-256	0.6120	0.4216	0.6297	0.4537	0.6545	0.4791	0.6436	0.3765	0.5499	0.4338	0.6050	0.4715
BOOST-256	0.6499	0.4553	0.6557	0.4563	0.6543	0.4562	0.6582	0.4331	0.7036	0.4904	0.6258	0.4637
L2-Net-HP-512	0.7265	0.5245	0.7398	0.5628	0.7271	0.4919	0.6848	0.4982	0.7342	0.5258	0.7371	0.5306
BEBLID-512	0.7914	0.5482	0.7259	0.5182	0.7807	0.5599	0.8169	0.5394	0.8105	0.5884	0.7615	0.5500
LBP+HOG-64	0.8327	0.5572	0.8039	0.5655	0.9249	0.5289	0.8360	0.5042	0.8392	0.4837	0.8080	0.6207
DT-CWT+HOG-63	0.8413	0.6523	0.8323	0.6711	0.8196	0.6993	0.8905	0.6477	0.8768	0.6029	0.8628	0.6163
DITF-270	0.9187	0.8041	0.9388	0.7667	0.9142	0.7281	0.8921	0.8258	0.9668	0.7740	0.9117	0.8135

**Figure 19:**
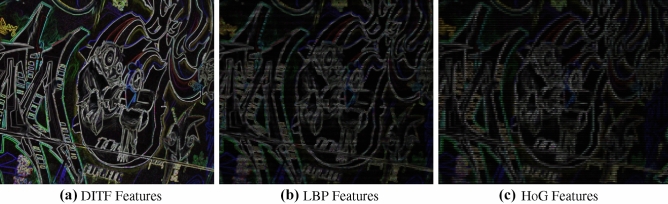
Feature extraction of DITF, LBP and HoG features.

**Figure 20:**
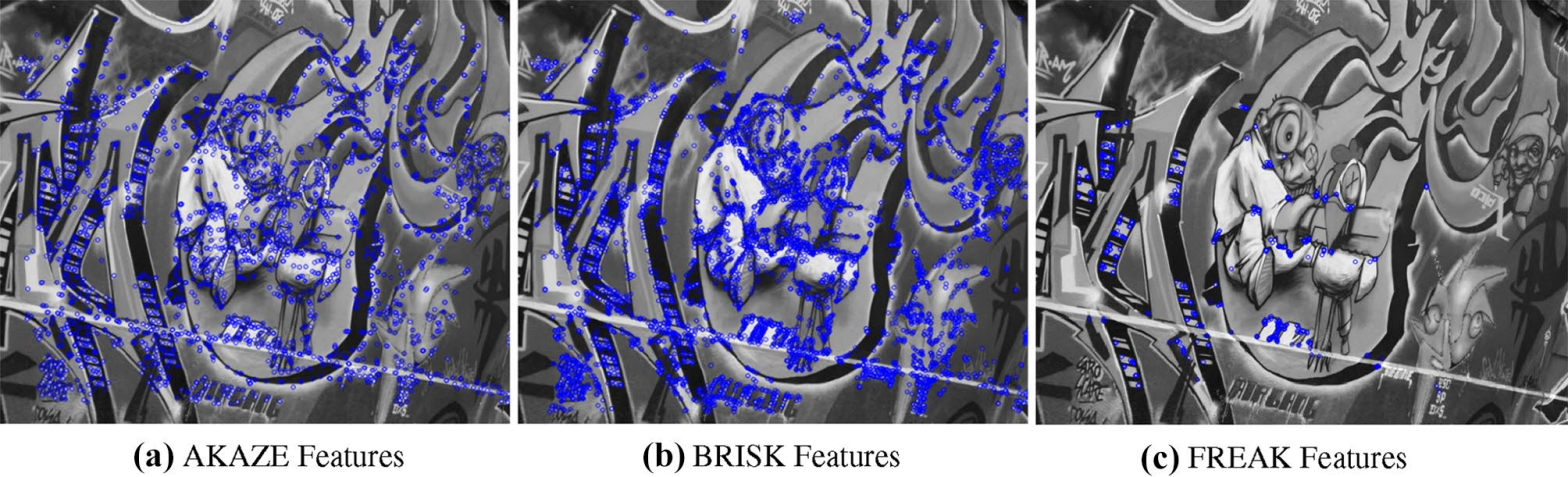
Feature extraction of AKAZE, BRISK and FREAK features.

In Table 1, the metrics mean average precision (mAP) and mean average recall (mAR) of the DITF method are presented and compared with other feature description algorithms, namely, BEBLID^25^, BOOST^26^, BRIEF^27^, AKAZE^28^, FREAK^12^ and BRISK^29^. This metric precision and recall quantify the quality of the feature extraction model. The dimension of DITF model depends on K,ll,R as mentioned in proposed model. The size of filter is 6*5 and the classes of feature vector is 9 then the dimension of DITF is 270. Basically, precision is used to measure the repetition or retrieval of the feature between reference and query image. The correct matches and predicted matches in the numerator and denominator of the Eqs. (13) and (12) are obtained from the repeatability score calculated in the previous sections. The higher the precision is a greater number of features are matched. In a similar fashion, the higher values of recall show better performance. Recall represents the correct matches of the query image has been measured from the correspondences of two images. DITF model outperforms the existing models. The second and third high values are scored by recent descriptors named as DT-CWT+HOG and LBP+HOG. These parameters are calculated as per the following equations:

12 $$\begin{aligned}{} & {} \text {Precision} = \dfrac{\text {correct matches}}{\text {predicted matches}} \end{aligned}$$

13 $$\begin{aligned}{} & {} \text {Recall} = \dfrac{\text {correct matches}}{\text {correspondence}} \end{aligned}$$

For the performance analysis of DITF model, we have included three variety of feature extraction for the validation and analysis. The Existing descriptors are compared with DITF in Figs. 19 and 20. LBP extract the texture information of the image and it’s shown in Fig. 19b. HOG features are illustrated in Fig. 19c, which provides the shape of the image. BRISK, and FREAK absorb the corner features. AKAZE extract blob feature in image. From the comparison analysis, BRISK has dense feature extraction than AKAZE descriptors. The features extracted from FREAK are deficient due to the fact that it is not associated with its own detector. The corner feature extraction has the scarcity in robustness. From this comparison, DITF model extract the maximum number of features by detecting the edge and corner of the image irrespective of transformation.

## Conclusion and future scope

In this article, we proposed a DITF based feature descriptor to improve the robustness against view-point variation, illumination changes and blur variation using Tri-ocular, Bi-ocular and Dia-ocular filter. It is verified that the DITF algorithm outperforms even when the images are of low resolution. It is noted that despite the low resolution, the algorithm extracted features with the repeatability scores of 100%, 100% and 99 % for the transformation, including, light variation, blur variation and view point variations respectively. We compared the DITF model with existing descriptors and verify that it achieved a significant percentage of improvement in precision and recall, when a few images from the data base is considered for the analysis. It is verified that the directional intensification with the help of tri filters can increase the robustness. However, the algorithm may not perform well when the image undergoes occlusion and scaling. These analyses open up a scope to address these issues in the future research.

## Data Availability

The datasets generated and/or analysed during the current study are available in the [AFFINE COVARIANT FEATURES] repository, [https://www.robots.ox.ac.uk/] and [SIPI IMAGE DATABASE-MISC], [http://sipi.usc.edu/database/].The DITF model can be re-generated using the following link (github.com/Johnchristopherclement/Directional-Intensified-Feature-Descriptor).
